# Interferon Regulatory Factors as a Potential Therapeutic Target for Neuroinflammation: A Focus on Alzheimer’s Disease

**DOI:** 10.3390/ijms26072906

**Published:** 2025-03-23

**Authors:** Xing Fan, Weikang Diao, Hao Wang, Xiaomin Yin, Wei Qian

**Affiliations:** Department of Biochemistry and Molecular Biology, School of Medicine, Nantong University, Nantong 226001, China; xingfan@ntu.edu.cn (X.F.); diaoweikang0210@163.com (W.D.); 13303474876@163.com (H.W.); yinxiaomin@ntu.edu.cn (X.Y.)

**Keywords:** interferon regulatory factors, neuroinflammation, Alzheimer’s disease, therapeutic targets

## Abstract

Interferon Regulatory Factors (IRFs) are critical modulators of immune and inflammatory responses, yet their roles in Alzheimer’s disease (AD) and other neurodegenerative disorders remain incompletely understood. While IRFs are recognized for their regulatory functions in neuroinflammation, microglial activation, and neuronal survival, their dual roles as both drivers of pathological inflammation and mediators of neuroprotective pathways underscore a sophisticated regulatory paradox in neurodegenerative disorders. This review aims to synthesize current evidence on IRF-mediated neuroinflammation in AD and related diseases, focusing on the multifaceted functions of key IRF family members, including IRF1, IRF3, and IRF7. We critically evaluate their divergent roles: IRF1 and IRF3, for instance, exacerbate neuroinflammatory cascades and amyloid-beta (Aβ) pathology in AD, whereas IRF7 may paradoxically suppress inflammation under specific conditions. Additionally, we explore IRF dysregulation in Parkinson’s disease, multiple sclerosis, amyotrophic lateral sclerosis, and Huntington’s disease, emphasizing shared and distinct mechanisms across neurodegenerative disorders. Restoring IRF balance through genetic manipulation, small-molecule inhibitors, or microbiome-derived modulators could attenuate neuroinflammation, enhance Aβ clearance, and protect neuronal integrity. Ultimately, this work provides a framework for future research to harness IRF signaling pathways in the development of precision therapies for AD and other neurodegenerative diseases.

## 1. Introduction

Dementia has become a health emergency of global concern, with 55.2 million people reportedly suffering from dementia today, and the number is forecast to reach 78 million in 2030 [1]. With the gradual acceleration of the aging process of the population, the financial burden of families with dementia will increase rapidly in the future, especially in low- and middle-income countries, which is expected to account for 65% of the global economic burden based on the value of a statistical life (VSL) by 2050, up from just 18% in 2019 [2]. Alzheimer’s disease (AD), one of the predominant forms of dementia, is the fifth leading cause of death among Americans aged 65 and older [3]. The etiology of AD is complex and diverse, and it may be caused by the joint action of multiple factors such as aging, genetics and the environment. Similarly, the understanding of the pathological mechanism of AD involves several hypotheses, including the cholinergic, the amyloid, the tau protein, the inflammation hypothesis, etc. [4]. Neuroinflammation may persist throughout the entire course of neurodegenerative diseases, intertwining with protein aggregates to exacerbate disease progression [5]. Currently, numerous therapeutic approaches targeting specific inflammatory signals are under clinical investigation. However, several key challenges remain in inflammation-targeted therapy, including the precise timing of signal transduction, cell-specific targeting, and the selection of appropriate molecular targets, all of which are crucial for minimizing the detrimental effects of inflammation while preserving its beneficial aspects [4,5].

Interferon Regulatory Factors (IRFs) constitute a comprehensive category of transcription factors initially identified as regulators of type I interferon (IFN-I) and IFN-responsive genes and can profoundly influence the immune system, impacting both physiological and pathological processes [6]. The IRF family comprises nine members in mammals, designated as IRF1 through IRF9 [7]. All IRFs feature a highly conserved N-terminal DNA-binding domain (DBD), comprising around 120 amino acids that constitute a helix-turn-helix motif, which is essential for the identification of specific DNA sequence elements (A/GNGAAANNGAAACT), known as the ISRE, present in the promoters of genes for IFN-I, IFN-III, and IFN-stimulated genes (ISGs) [6,8]. The IRF family is implicated in nearly all human cancers, where its members can play protective or detrimental roles. Some IRF members act as double-edged swords in cancer development, exhibiting both tumor-suppressive and oncogenic functions [6].

The objective of this review is to elucidate the roles of the IRF family in neuroinflammation and neuropathic pain, as well as its impact on the pathogenesis of neurodegenerative diseases, particularly AD. We aim to explore therapeutic strategies targeting the IRF family, comprehensively evaluate its functions and influence in AD, and provide novel and effective insights for the treatment of brain disorders.

## 2. AD and Neuroinflammation

Neuroinflammation, driven by different immune components such as activated glia, cytokines, chemokines, and reactive oxygen species, can regulate every step of adult neurogenesis, including cell proliferation, differentiation, migration, survival of newborn neurons, maturation, synaptogenesis, and neurogenesis [9]. It therefore plays a critical role in the etiology and pathology of numerous neurological disorders, including AD, multiple sclerosis, Parkinson’s disease, epilepsy, and stroke [10]. Neuroinflammation can be both detrimental, leading to neuronal damage, and beneficial, promoting tissue repair. This duality complicates the research and therapeutic approaches to neuroinflammation [10].

In AD, chronic neuroinflammation emerges as a central driver for the neurodegenerative process due to the orchestrated interaction among multiple cell types including but not limited to microglia, astrocytes, and neurons [11]. Neuroinflammation is not merely a passive response to emerging senile plaques and neurofibrillary tangles but actively contributes to pathogenesis to an extent that may be equal to or greater than that of the plaques and tangles themselves [12]. Currently, the neuroinflammation treatment strategies for AD primarily focus on the following aspects. The first is modulating neuroinflammatory pathways, including pharmacological interventions of pathways such as NF-κB, NLRP3, triggering receptor expressed on myeloid cell 2 (TREM2), and cyclic GMP-AMP synthase-stimulator of interferon genes (cGAS-STING) [13]. These measures are designed to intervene in the regulation of cytokine, chemokine, interferon, and interleukin release and accumulation by microglia and neurons [14]. NF-κB is a transcription factor that promotes inflammation by upregulating the expression of pro-inflammatory cytokines and chemokines. Inhibiting key steps in its activation, including nuclear translocation, DNA binding, phosphorylation of NF-κB, and degradation of IκB, may represent a promising therapeutic strategy for treating inflammatory conditions [13]. The activation of the NLRP3 inflammasome may contribute to early pathological events in AD, such as memory impairment and Aβ deposition. NLRP3 inhibitors, including MCC950, JC124, VX-765, and OLT1177, reduce NLRP3 activity through distinct mechanisms, thereby alleviating inflammation and demonstrating improvements in cognitive function and reductions in Aβ deposition in animal models [15]. TREM2, a transmembrane receptor, plays a critical role in Aβ clearance and in responding to brain injury and inflammation. Through multiple signaling pathways, TREM2 modulates various biological functions of microglia, including promoting microglial proliferation, inhibiting apoptosis, regulating inflammatory responses, and modulating lipid metabolism [16]. In AD models, the absence of cGAS protects against cognitive impairment and ameliorates Aβ pathology and neuroinflammation. To date, no inhibitors targeting the cGAS-STING pathway have been approved for AD treatment; however, compounds such as H-151 have demonstrated mitigating effects on AD pathology in both cell culture models and animal studies [17].

Second, the regulation of cell survival and death modes plays a crucial role in cellular responses. For instance, endoplasmic reticulum (ER) stress activates the unfolded protein response (UPR), which triggers rapid protein kinase reactions via multiple transducers, subsequently altering the expression of numerous target genes. Additionally, the UPR can activate the apoptotic cell death program during prolonged stress. Studies have shown that ER stress may also generate signals to warn neighboring cells and trigger inflammatory responses to counteract increased tissue injury [18]. Autophagy activation can inhibit inflammatory responses by degrading inflammasomes or pro-inflammatory cytokines and enhancing immune system function. For example, autophagy activators such as resveratrol can inhibit inflammation through multiple mechanisms and reduce Aβ-induced neurotoxicity [19]. Different modes of cell death, including apoptosis, necrosis, pyroptosis, and ferroptosis, can serve as drivers of inflammation in the central nervous system (CNS). These various forms of cell death may independently or collectively contribute to neuronal loss [20].

Third, the development of targeted therapies for Aβ and tau pathologies has garnered significant attention [21]. Currently, several drugs targeting Aβ have received accelerated approval from the FDA. For tau-related disease-modifying therapies, approaches include mediators that regulate tau post-translational modifications, anti-tau immunotherapies (both active and passive), tau aggregation inhibitors, microtubule stabilizers, and gene therapies. Although no tau-targeting drugs have yet been approved by the FDA, several agents aimed at tau post-translational modifications and propagation have advanced to clinical trials [21].

Additionally, another important area of research involves targeting molecular targets such as APOE, SR-A, and CD33 receptors, as well as non-coding RNAs and epigenetic mechanisms. The human APOE gene has three alleles (ε2, ε3, and ε4), encoding three isoforms of the APOE protein. Among these, APOE2 is associated with a protective effect, while APOE4 is linked to an increased risk of AD. Reducing APOE4 expression may be beneficial for mitigating AD pathology; however, its efficacy and cell-specific effects require further investigation [22]. Non-coding RNAs influence gene expression and neuroinflammatory processes by interacting with multiple targets [23]. Epigenetic modifications play a crucial role in AD pathology and cognitive function through their involvement in processes such as APP metabolism, Aβ formation, tau phosphorylation, oxidative stress responses, apoptosis, and inflammatory responses [24].

Finally, lifestyle modifications also play a crucial role. These include increasing physical activity [25]; adopting a dietary pattern low in animal products and rich in anti-inflammatory and low glycemic load foods [26]; consuming more natural products such as fruits, vegetables, herbs, nuts, tea, and macro fungi [27]; regulating the gut microbiota through probiotic supplementation [28]; and other related interventions.

## 3. IRFs and Neuroinflammation in AD

### 3.1. IRF1

**Figure 1:**
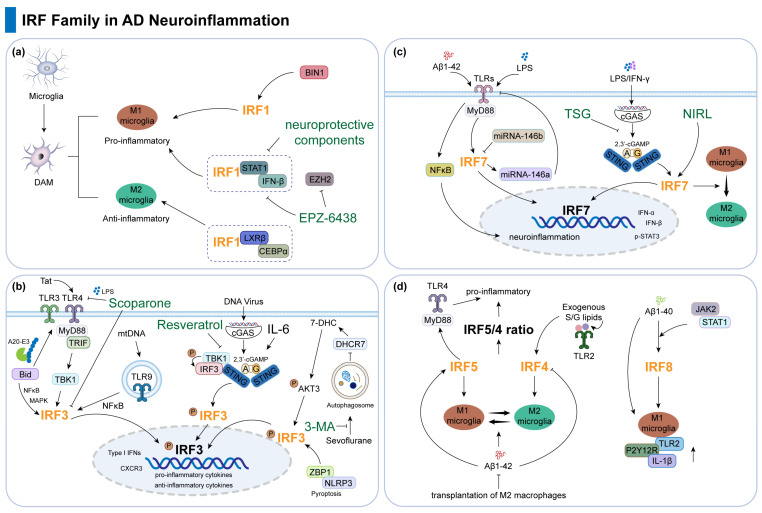
The regulatory effects and molecular mechanisms of the IRF family in AD-associated neuroinflammation. (**a**) The role of IRF1 in the phenotypic transformation of microglia. (**b**) IRF3 modulates neuroinflammation and AD progression via TLR/STING signaling pathways and the interplay between autophagy and pyroptosis. (**c**) IRF7 modulates AD-associated neuroinflammation via the TLR/STING-miRNA interaction network and microglial M1/M2 phenotypic polarization. (**d**) The regulatory network of IRF4, IRF5, and IRF8 in microglial polarization.

The role of the IRF family, especially IRF1, 3, and 7, in neuroinflammation has now been widely reported in AD as well as in neurodegenerative diseases. Microglia are intrinsic immune cells of the CNS, and in AD, steady-state microglia are transformed into disease-associated microglia (DAM) profiles; there are different subpopulations of DAM, such as pro-inflammatory and anti-inflammatory [29]. IRF1 not only plays a key role in the pro-inflammatory DAM state but also positively regulates the anti-inflammatory DAM gene and the homeostatic genes and participates in the transcriptional regulatory network of microglia together with other transcription factors such as LXRβ and CEBPα [29]. Bridging Integrator 1 (BIN1), the second most important genetic risk factor for late-onset AD (LOAD), regulates pro-inflammatory and neurodegeneration-related activation responses in microglia. When BIN1 expression is aberrant, it leads to altered IRF1 expression, which in turn affects inflammation-related processes such as cytokine production and release in microglia in response to inflammatory stimuli [30]. EPZ-6438, a histone methyltransferase (EZH2) inhibitor, controls important inflammatory gene targets by inhibiting the transcription of microglia activation-related genes, such as IRF1, IRF8, and the levels of signal transducer and activator of transcription (STAT) 1 [31]. In addition, some neuroprotective components such as phytoestrogens [32], foods rich in biologically active phytochemicals such as pheophytin α (PP) and chlorophyll a (CP) [33], and the plant biosynthesized open-chain flavonoid chalcone [34] act as anti-inflammatory agents for the treatment of neurodegenerative diseases by inhibiting the activation of signaling pathways, such as IRF1, STAT1, and IFN-β, and the release of related pro-inflammatory factors (Figure 1a; Table 1).

### 3.2. IRF3

In normal tissues, IRF3 expression exhibits a highly cell-type-specific and organ-specific pattern. It is prominently expressed in certain epithelial cells of the lungs, liver, kidneys, and other organs but is either low or absent in immune cells and brain parenchyma. In CNS diseases, except for HIV encephalitis patients with abnormal IRF3 immunoreactivity in multinucleated giant cells in the brain, brain samples from other diseases are not significantly different from normal [59]. Notably, IRF3 mRNA expression is significantly elevated in the brains of AD patients and correlates positively with Toll-like receptor-3 (TLR3) mRNA expression as well as plaque and tangle scores in AD brains. Furthermore, IRF3 may exert an anti-inflammatory effect in microglia via activation of the AKT/PI3 signaling pathway [35].

TLRs are transmembrane proteins primarily involved in immune responses and are expressed by several immune and non-immune cells within the CNS. Signaling of TLRs affects the core of AD changes, including synaptic plasticity, microglial activity, tau phosphorylation, and inflammatory responses [60]. In neurodegenerative diseases, IRF3 serves as a key transcription factor in TLR signaling pathways, responsible for activating the expression of type I interferons and other inflammation-related genes. The BH3-interacting domain death agonist (Bid) plays a pivotal role in modulating downstream inflammatory signaling mediated by TLR3 and TLR4. Bid deficiency results in enhanced interaction with the A20-E3 ubiquitin ligase, leading to inhibition of downstream signaling cascades involving NF-κB, MAPK, and IRF3, thereby attenuating the inflammatory response [36]. The HIV trans-activator of transcription (Tat) protein activates the TLR4 signaling pathway, leading to the subsequent activation of TANK-binding kinase 1 (TBK1) and IRF3. This cascade ultimately results in the upregulation of CXC chemokine receptor 3 (CXCR3) expression, thereby promoting monocyte transmigration across the blood–brain barrier (BBB) [37]. Scoparone (6,7-dimethoxycoumarin), one of the major active natural bioactive compounds in the inner shell of chestnut (Castanea crenata), alleviates neuroinflammation by protecting against LPS-induced inflammation through TLR4 signaling via TIR-domain-containing adapter-inducing interferon-β (TRIF) and myeloid differentiation primary response gene 88 (MyD88) adaptor proteins, leading to reduced IRF3 activation [38]. Moreover, mitochondrial DNA (mtDNA) fragments can bind to TLR9, which in turn activates the NF-κB signaling pathway and IRF3. This activation triggers the expression of interferon-stimulated genes and enhances the interferon response [39].

The cytosolic DNA sensor cGAS-STING pathway was initially thought to be activated primarily by viral DNA [61]. In neurodegenerative diseases, the activation of the STING signaling pathway regulates the expression of type I interferons and other inflammation-related genes by influencing the phosphorylation and nuclear translocation of IRF3, thereby participating in the disease-related neuroinflammatory process [62]. Research demonstrates that 2′3′-cyclic guanosine monophosphate-adenosine monophosphate (cGAMP), a high-affinity and selective STING agonist, significantly improves cognitive function in AD mice, reduces Aβ plaque burden, decreases neuronal apoptosis, and ameliorates AD-related pathological changes by activating the cGAMP-STING-IRF3 signaling pathway [40]. cGAMP also exerts its anti-inflammatory effects by promoting the phosphorylation and activation of IRF3 via downstream TBK1 activation, thereby regulating the expression of multiple anti-inflammatory and immune-related genes [41]. In the model of neuroinflammation and cognitive dysfunction induced by chronic cerebral hypoperfusion (CCH), the natural polyphenolic compound resveratrol alleviates neuroinflammation, improves cognitive function, and protects neurons and white matter structure by inhibiting the STING/TBK1/IRF3 signaling pathway [42]. Furthermore, Interleukin-6 (IL-6) deficiency alleviates neuroinflammation, reduces Aβ deposition, and improves cognitive function by inhibiting the STAT3-cGAS-STING pathway, thereby providing a potential therapeutic target for AD treatment [43].

Autophagy plays a crucial role in clearing misfolded protein aggregates, damaged mitochondria, and their generated ROS and in degrading the NLRP3 inflammasome or its components [63]. Hippocampal autophagy is crucial for memory formation [64]. The inhaled anesthetic sevoflurane activates IRF3 via the autophagy pathway, which includes the degradation of dehydrocholesterol-reductase-7 (DHCR7) and subsequent activation of AKT3. The autophagy inhibitor 3-MA can mitigate sevoflurane-induced IRF3 activation and cytokine expression, thereby alleviating cognitive impairment [44]. NLRP3 inflammasome-mediated pyroptosis may contribute to the progression of AD-related memory loss [63]. Z-DNA binding protein 1 (ZBP1), a sensor host gene, is abnormally upregulated in AD. Silencing ZBP1 suppressed cell injury and pyroptosis of AD neurons and improved the cognitive function of AD rats via inhibiting IRF3 [45]. Conversely, ZBP1 as a target of IRF3 is relevant across various neuroinflammatory disorders. In LPS-induced inflammatory responses, IRF3 exerts its effects by modulating cytokine production, sickness behavior, myeloid cell infiltration, inflammasome activation, and the expression of genes such as ZBP1 [65].

In addition, in disease models associated with AD development, IRF3 can regulate interferon production via multiple pathways and contribute to neuroinflammation. In the context of herpes simplex virus type 1 (HSV-1) infection, the stress hormone corticosterone (CORT) inhibits IRF3 phosphorylation by modulating the glucocorticoid receptor (GR), thereby reducing IFN-β production and suppressing innate immune responses [66]. In a sleep disruption model, protein kinase RNA-activated (PKR) binds to IRF3, promoting its activation and nuclear translocation, thus regulating the expression of interferons and other antiviral genes [67] (Figure 1b; Table 1).

### 3.3. IRF7

Analysis of postmortem brain tissues, including hippocampal and temporal cortex samples, from AD patients and non-demented controls revealed that the mRNA levels of IRF7, MED23, IL28B, and IFN-α were significantly reduced in the hippocampus and temporal cortex of most AD patients [46]. Furthermore, analysis of brain tissues from carriers of the TREM2 R47H variant, sporadic AD patients, and normal controls demonstrated that the TREM2 R47H variant is strongly associated with an increased risk of AD and can exacerbate neuroinflammation in the brains of AD patients. Specifically, the TREM2 R47H variant promotes the activation of IRF7, thereby enhancing type I interferon responses and accelerating the neurodegenerative process in AD [49]. Several studies have also indicated that during CNS injury, IRF7 is upregulated in microglia and plays a role in mediating inflammatory responses [47,48].

Analogous to IRF3, IRF7 also serves as a critical transcription factor in the TLR and STING signaling pathways. The type I interferon response of neurons to soluble amyloid-β (Aβ) is primarily mediated by TLRs and relies on Myd88 and IRF7 signaling. Knockdown of IRF7 effectively blocks the expression of IFN-α, IFN-β, and p-STAT3 induced by Aβ1-42 and LPS [50]. In miRNA regulation, miR-146b-deficient mice exhibited a significant increase in IRF7 expression, which may subsequently upregulate miR-146a, thereby further inhibiting TLR4 expression and reducing NF-κB activation and neuroinflammatory responses [51]. Tetrahydroxy stilbene glucoside (TSG), a natural active ingredient derived from the Chinese herb polygonum multiflorum, reduces the transcriptional levels of factors such as IRF7 in LPS/IFN-γ-stimulated microglial cells by inhibiting the cGAS-STING pathway. This inhibition, in turn, decreases neuroinflammation and may have potential therapeutic effects in AD [52]. In addition, in a study examining the therapeutic effects of near-infrared light therapy (NIRL) on postoperative neurocognitive disorder (PND) in elderly mice, NIRL was found to upregulate IRF7 expression. This upregulation promotes the transition of microglia from the pro-inflammatory M1 phenotype to the neuroprotective M2 phenotype, thereby reducing brain damage and improving cognitive function in PND mice [53] (Figure 1c; Table 1).

### 3.4. Other IRFs

IRF5 facilitates the polarization of classically activated (M1) phenotype microglia, whereas IRF4 drives the polarization of alternatively activated (M2) phenotype microglia. In AD models, elevated levels of IRF5 contribute to an increased proportion of M1-type microglia, while reduced levels of IRF4 are associated with a decreased proportion of M2-type microglia [54]. Aβ1-42 can alter the polarization state of microglia, leading to an increased IRF5/4 ratio. The transplantation of M2 macrophages can modulate this ratio, thereby alleviating inflammation and improving cognitive impairment. These findings suggest that IRF4 plays a critical role in regulating immune responses and promoting neuroprotection in AD [55]. Studies on microbiome-derived bacterial lipids have also confirmed the protective role of IRF4. Exogenous supplementation of serine–glycine (S/G) lipids, which act as TLR2 ligands, can restore IRF4 gene expression levels, thereby suppressing excessive pro-inflammatory responses [56]. Conversely, down-regulating IRF5 in AD can alleviate the inflammatory response and enhance microglial phagocytosis and the degradation of Aβ [48]. In studies of learning and memory impairments caused by Borna disease virus (BoDV-1) infection, IRF5 promotes the expression of inflammatory factors via activation of the TLR4/MyD88 signaling pathway, thereby intensifying neuroinflammation and negatively impacting cognitive function [68].

In contrast to IRF7 [46,49], IRF8 expression in microglia is upregulated in AD brains and co-expressed with other markers indicative of microglial response to Aβ such as Iba1, CD68, and HLA-DR. This suggests that IRF8 participates in the microglial activation associated with AD pathology. In the AD brains of TREM2-R47H carriers, the expression of microglial genes, including IRF8, HLA-DRA, and AIF1, is significantly reduced, indicating that IRF8 may play a critical regulatory role in TREM2-related AD pathogenesis [57]. Simultaneously, IRF8 expression was significantly elevated in the brains and microglia of AD transgenic Tg2576 mice. Aβ1-40 promotes IRF8 expression at the transcriptional level, with the JAK2/STAT1 pathway mediating this increase. Silencing IRF8 via siRNA eliminated the upregulation of typically activated microglial genes, such as TLR2, P2Y12R, and IL-1β induced by Aβ1-40, while overexpression of IRF8 exacerbated the expression of these proteins [58] (Figure 1d; Table 1).

Members of the IRF family display considerable functional heterogeneity and regulatory complexity in the neuroinflammatory processes associated with AD. Their mechanisms of action share common features while also exhibiting unique target specificity. As a central regulatory hub, IRF1 sustains immune homeostasis by simultaneously promoting the activation of pro-inflammatory microglia and facilitating the co-expression of anti-inflammatory genes. Nevertheless, its regulatory network can be disrupted by genetic risk factors, such as BIN1 abnormalities, resulting in inflammatory dysregulation [29,30]. In contrast, the “double-edged sword” characteristic of IRF3 is particularly pronounced. On the one hand, IRF3 exacerbates Aβ-related inflammatory responses through the TLRs/cGAS-STING pathway [36,37,38,39,40,41,42,43,62]; on the other hand, it indirectly confers neuroprotection via the AKT/PI3K pathway or autophagy [35,44]. This indicates that its functional outcomes are highly contingent upon the integration of microenvironmental signals and its subcellular localization. The regulatory plasticity of IRF7 further underscores the dynamic adaptability of the IRF family members. While it promotes the type I interferon pro-inflammatory cascade via the TLR/STING pathway [50,52], its effects can be reversed by miRNA-mediated negative feedback (e.g., the miR-146b-IRF7 axis) or physical interventions (e.g., NIRL), thereby inducing microglial polarization toward the M2 phenotype [51,53]. This highlights the significant potential for epigenetic and external factors to deeply remodel its functional outcomes. It is worth noting that IRF4 and IRF5 antagonistically regulate microglial polarization (M1/M2), thereby directly determining the inflammatory phenotype. The imbalance in their ratio, such as the Aβ-induced increase in IRF5/IRF4, serves as a pathological hallmark of AD [54,55]. In contrast, IRF8 specifically mediates Aβ-dependent microglial activation via the TREM2-JAK2/STAT1 axis, highlighting its potential as a unique therapeutic target for AD associated with TREM2 mutations [57,58].

Therefore, the bidirectional regulatory characteristics of IRF family members are influenced by multiple factors, such as the integration of microenvironmental signals (e.g., the pro-inflammatory TLR/STING pathway and the anti-inflammatory AKT/PI3K pathway), cell type specificity (e.g., differences in signal regulation in neurons and microglia), genetic variations, and epigenetic modifications, among others.

## 4. IRFs and Neuroinflammation in Neurodegenerative Diseases

### 4.1. Parkinson’s Disease

Parkinson’s disease (PD) is the second most common neurodegenerative disorder after AD. As one of the most devastating neurodegenerative disorders, PD is associated with degeneration of dopaminergic neurons and the accumulation of α-synuclein, which results in motor disability and cognitive decline [69,70]. Neuroinflammation has emerged as a mechanism involved at the initiation and development of PD. It is a complex network of interactions comprising immune and non-immune cells in addition to mediators of the immune response [71].

**Figure 2:**
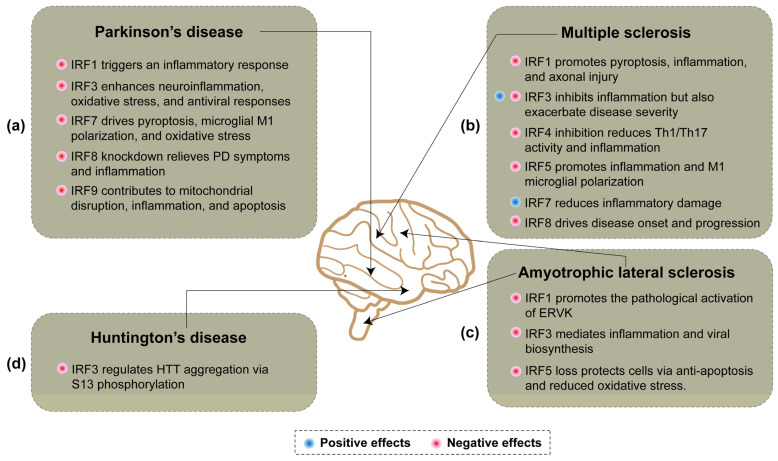
The potential role of IRFs in other neurodegenerative diseases. (**a**) IRF1, IRF3, IRF7, IRF8, and IRF9 negatively regulate Parkinson’s disease. (**b**) IRF1, IRF4, IRF5, and IRF8 present negative roles; IRF7 plays protective roles; and IRF3 plays dual roles in multiple sclerosis. (**c**) IRF1, IRF3, and IRF5 negatively regulate amyotrophic lateral sclerosis. (**d**) IRF3 negatively regulates Huntington’s disease.

Multiple members of the IRF family play crucial roles in neuroinflammation associated with PD (Figure 2a). During the inflammatory process in PD, microglial activation leads to the upregulation of key transcription factors such as NF-κB, IRFs, and AP-1, thereby triggering an inflammatory response [72]. Glucocorticoids (GCs) are a class of clinically used anti-inflammatory drugs, and when their secretion increases, their receptor GR will be activated. GR inhibits the transcriptional activity of key transcription factors such as IRFs, NF-κB, and AP-1, thereby suppressing the inflammatory response and exerting neuroprotective effects [72]. A study on PTEN-induced putative kinase 1 (PINK1) has demonstrated that wild-type PINK1 inhibits the VCAM-1 promoter by suppressing the transcriptional activity of IRF1, whereas PINK1G309D, a homozygous mutation of PINK1, enhances VCAM-1 promoter activity by upregulating IRF1 transcription [73]. Consequently, this leads to pro-inflammatory effects and increased monocyte adhesion to brain endothelial cells [73].

In addition to IRF1, IRF3 also plays a crucial role in PINK1-mediated regulation [74]. PINK1 promotes the activation of IRF3 and NF-κB by inhibiting the Parkin-mediated K48-linked ubiquitination and degradation of TRAF3, thereby enhancing the production of type I interferons and pro-inflammatory cytokines. Furthermore, PINK1 interacts with Yes-associated protein 1 (YAP1) and inhibits the formation of the YAP1/IRF3 complex, thereby relieving the YAP1-mediated suppression of the cellular antiviral response and promoting RLR signaling pathway-mediated innate antiviral immunity [74]. Several natural plant extracts, such as curcumin [75] and silibinin [76], exhibit protective effects against neuroinflammation and ameliorate depression- and anxiety-like behaviors in PD model mice. Curcumin pretreatment inhibits MPP+-induced IRF3 activation via the TLR4 pathway, thereby reducing IRF3 phosphorylation and subsequently decreasing the production of inflammatory mediators. This exerts a protective effect against neuroinflammation and oxidative stress [75]. Silibinin treatment significantly reduced IRF3 levels in the hippocampus of MPTP-treated mice and decreased STING and IFN-β levels. Additionally, it downregulated the expression of downstream inflammatory factors, including caspase1, IL-1β, and TNFα. These findings suggest that silibinin may protect hippocampal neurons by inhibiting the STING-IRF3 signaling pathway and mitigating inflammatory responses [76]. Finally, absent in melanoma 2 (AIM2), an essential inflammasome protein, can reduce cGAS-mediated antiviral-related inflammation by inhibiting AKT-IRF3 phosphorylation [77].

Regarding IRF7, existing studies demonstrate that IRF7 functions as a transcription factor to directly regulate the expression and activation of NLRP3 in the pyroptosis pathway. Inhibition of the TLR4/TAK1/IRF7/NLRP3 signaling axis can reduce pyroptosis in dopaminergic neurons, thereby slowing the progression of PD [78]. In addition, the activation of IRF7 is closely associated with M1 polarization and the inflammatory response in microglia. Inhibiting IRF7 or its upstream molecules can significantly attenuate the inflammatory response and promote microglial polarization toward the M2 phenotype, specifically via the cGAS-STING-IRF7 signaling pathway [79]. The deubiquitinase OTUB1 can stabilize IRF7 through deubiquitination, thereby enhancing IRF7’s binding to the promoter of NADPH oxidase 4 (NOX4) and promoting its expression. This process exacerbates oxidative stress injury (OSI) and inflammatory responses [80].

Currently, there are limited reports on the roles of IRF8 and IRF9 in PD. Studies have demonstrated that knocking down IRF8 can alleviate behavioral deficits in MPTP-induced PD mouse models, increase the dopamine content, reduce inflammatory cytokine levels, and inhibit the activation of the AMPK/mTOR signaling pathway [81]. The expression of IRF9 is upregulated in PD models and, in conjunction with the transcription factor NFATc2, contributes to the regulation of IFN-I signaling [82]. As a target gene of miR-20a-5p, IRF9 contributes to MPP+-induced mitochondrial disruption, inflammation, and cell apoptosis. Moreover, IRF9 hinders the improvement of miR-20a-5p overexpression on MPP+-induced neurotoxicity. Furthermore, the decrease in p-P65 levels induced by miR-20a-5p mimic is significantly reversed by IRF9 overexpression [83].

### 4.2. Multiple Sclerosis

Multiple sclerosis (MS) is a disease of the CNS characterized by chronic inflammation and demyelination. It is the most common autoimmune disease in the brain and the leading cause of non-traumatic neurological disability in young adults [84,85]. The IRF family, particularly IRF1, IRF3, and IRF7, plays a crucial role in mediating neuroinflammation in MS (Figure 2b). IRF-1 plays a multifaceted role in MS and its animal model, experimental autoimmune encephalomyelitis (EAE). First, IRF-1 exacerbates inflammatory demyelination and axonal injury by promoting oligodendrocyte pyroptosis through the upregulation of caspase1 expression [86]. Furthermore, IRF-1 independently regulates inflammatory demyelination and disease severity in CNS glial cells via the modulation of caspase1 expression [87]. In EAE, IRF-1 enhances the expression of pro-inflammatory and pro-apoptotic molecules within the CNS, linking inflammation to tissue injury processes. The absence of IRF-1 expression provides partial protection against disease progression [88]. Low IRF-1 expression is associated with abnormal STAT1 phosphorylation, leading to the insufficient expression of ISGs, which impacts the pathological mechanisms of MS [89]. Single nucleotide polymorphisms (SNPs) in the IRF-1 gene are also linked to increased susceptibility to MS [90]. In B cells from MS patients, the downregulation of IRF-1 and CXCL10 expression, potentially mediated by upregulated hsa-miR-424, results in a pro-survival state of B cells, contributing to MS pathology [91]. Targeting the IRF-1 signaling pathway may offer a novel therapeutic strategy for MS by protecting oligodendrocytes and myelin, thereby reducing axonal injury and achieving neuroprotection [92]. Phytoestrogens exert anti-inflammatory effects by inhibiting IRF-1 and pSTAT1 expression and reducing NO production and inflammatory cytokine release in microglia [32]. Isosorbide di-(methyl fumarate) (IDMF) alleviates inflammation and pyroptosis by inhibiting the IRF-1 pathway [93]. Finally, IFN-γ activates STAT-1 to regulate IRF-1 expression and function, influencing immune cell activities and immune response regulation, including its role in EAE [85].

IRF3 and IRF7 play equally critical roles in regulating neuroinflammation and immune responses in MS and its animal model EAE. IRF3 exerts a protective effect in MS by inhibiting neuroinflammation through the regulation of immunoregulatory miRNA in human astrocytes [94]. In EAE, IRF3 deficiency results in reduced disease severity and impaired transferability of EAE by IRF3-deficient cells, indicating its involvement in disease progression [95]. Furthermore, IRF3 activation is a key step in the STING pathway, which exacerbates neuroinflammation in neurodegenerative diseases, including MS, via the production of downstream factors such as IFN-β [62]. IRF3 also mediates anti-inflammatory effects in microglia via the PI3K/Akt pathway, promoting a shift from pro-inflammatory to anti-inflammatory states, which may have therapeutic potential [96]. Conversely, IRF7 plays an essential role in regulating CNS inflammation. Its deficiency exacerbates EAE symptoms and increases CNS infiltration, highlighting its protective function in autoimmune demyelination [97]. Elevated IRF7 expression in MS patients enhances endogenous IFN-like activity, potentially influencing the response to interferon-β therapy, although it does not directly predict long-term disease progression [98]. Moreover, IRF7-regulated type I interferon signaling inhibits CXCL13 production, thereby reducing inflammatory damage in MS [99]. During neuroinflammation, IRF7 is crucial for the function of plasmacytoid dendritic cells (pDCs) in the CNS, enabling the rapid production of type I IFNs and IL-12p40 in response to TLR9 stimulation, which is vital for modulating immune responses in EAE [100]. Collectively, these findings underscore the complex and multifaceted roles of IRF3 and IRF7 in MS pathogenesis, highlighting their potential as therapeutic targets for modulating neuroinflammation and immune responses.

Finally, other IRF family members such as IRF4, 5, and 8 are also involved in MS-mediated neuroinflammation. In EAE, inhibiting IRF4 reduces disease severity by decreasing Th1 and Th17 cell infiltration, differentiation, and pro-inflammatory cytokine expression, underscoring its role in regulating neuroinflammation [55]. Variations in the IRF5 gene are significantly associated with an increased risk of MS, potentially affecting immune regulation and contributing to autoimmune responses [101]. Furthermore, the histone deacetylase inhibitor Panobinostat alleviates EAE by suppressing the TLR2/MyD88/IRF5 signaling pathway, thereby reducing pro-inflammatory cytokine production and M1 microglial polarization, further highlighting the importance of IRF5 in neuroinflammation [102]. IRF8 is another critical member of the IRF family that is crucial in the pathogenesis of EAE. It is predominantly expressed in antigen-presenting cells (APCs) rather than T cells and facilitates disease onset and progression through multiple pathways [103]. Collectively, these findings underscore the complex and multifaceted roles of IRF family members in MS (Figure 2b).

### 4.3. Amyotrophic Lateral Sclerosis

Amyotrophic lateral sclerosis (ALS) is a fatal neurodegenerative disorder associated with the loss of both upper and lower motor neurons that leads to muscle weakness, paralysis, and eventually death [104]. The role of IRF family-mediated neuroinflammation in ALS is predominantly attributed to IRF3 (Figure 2c). Notably, the ubiquitin-binding protein optineurin (OPTN) has been extensively studied for its regulation of ALS via the IRF3 signaling pathway. Research indicates that wild-type OPTN can suppress IRF3 activation, whereas ALS-associated mutations (such as Q398X and E478G) lose this inhibitory function, leading to aberrant IRF3 activation [105]. This dysregulation can result in excessive IFN-β expression or apoptosis mediated by the Bax transcription factor [106]. Additionally, OPTN interacts with TBK1 to modulate the activation of IRF3 and IRF7, thereby influencing type I interferon production [107]. However, some studies suggest that OPTN may act as a positive regulator of the IRF3 signaling pathway; for instance, IRF3 signaling is impaired in bone marrow-derived macrophages (BMDMs) from OPTN-deficient mice upon TLR3/4 stimulation [108]. The role of TBK1 as an upstream effector molecule of IRF3 in ALS has also been extensively investigated. Exome sequencing of familial ALS patients revealed that specific ALS-associated mutations in TBK1, particularly p.G217R and p.R357X, impair its ability to phosphorylate IRF3, indicating a loss of TBK1 kinase activity [109]. However, these functional mutations do not significantly affect IRF3 expression or localization [110]. Furthermore, the autophagy receptor Ubiquilin 2 enhances TBK1 protein stability and phosphorylation levels, thereby promoting IRF3 phosphorylation and increasing the production of IFN1 and related pro-inflammatory cytokines [111]. Finally, Bid [36] and the human helicase senataxin (SETX) [112] exert both positive and negative regulation on IRF3, thereby modulating IRF3-mediated inflammatory and viral biogenesis.

Currently, there is limited literature on the role of IRF1-mediated neuroinflammation in ALS. Only a few studies have demonstrated that the nuclear translocation of IRF1 in ALS brain tissue is associated with the reactivation of endogenous retrovirus-K (ERVK), suggesting that IRF1 may contribute to the pathological activation of ERVK in ALS [113]. Additionally, a limited number of reports have focused on the role of IRF5: the loss of IRF5 in TDP-25 cells exerts a protective effect mainly by inhibiting apoptosis, regulating cell cycle arrest and alleviating oxidative stress, and IRF5 can mediate neuronal injury partly through the negative regulation of TBK1 [114] (Figure 2c).

### 4.4. Huntington’s Disease

Huntington’s disease (HD) is a monogenic, fully penetrant, progressive neurodegenerative disorder characterized by motor, cognitive, and psychiatric disturbances [115]. In HD, an expansion of a CAG repeat within exon 1 of the huntingtin (HTT) gene, which produces an HTT protein with an expanded polyglutamine (polyQ) repeat, leads to a progressive and fatal neurodegenerative pathology [116,117,118].

The role of IRF family-mediated neuroinflammation in HD remains largely unexplored. Limited evidence suggests that IRF3 may function as a critical transcription factor within the non-canonical IKK pathway, thereby modulating HD pathology. Specifically, inhibitor of nuclear factor kappa B kinase subunit beta (IKBKB) activates IRF3 via this non-canonical pathway. Following activation, IRF3 influences the aggregation propensity and pathological phenotypes of HTT by regulating its S13 phosphorylation state [119] (Figure 2d).

## 5. Conclusions

The emerging role of IRFs in the pathogenesis of AD and other neurodegenerative diseases, such as PD, MS, ALS, and HD, highlights their potential as a novel therapeutic target. The dysregulation of IRFs, particularly IRF1, IRF3, and IRF7, has been implicated in the neuroinflammatory processes and Aβ pathology that drive AD progression. By modulating IRF activity, it may be possible to attenuate neuroinflammation, reduce Aβ accumulation, and improve neuronal survival. However, the dual roles of IRFs in both protective and detrimental pathways necessitate a nuanced approach to therapeutic intervention. For instance, the “double-edged sword” nature of IRF1/IRF3 necessitates the precise modulation of pro-inflammatory pathways while preserving protective functions. The dynamic balance of IRF4/5 must be spatially regulated through targeted delivery mechanisms (e.g., M2 macrophage transplantation) or lipid signaling interventions [55,56]. Furthermore, the microenvironment-dependent actions of IRF7/IRF8 indicate that single-pathway inhibition alone may not suffice to reverse inflammatory cascades, and multi-target collaborative strategies (e.g., combining natural compounds such as TSG with gene editing) are required [52,58]. Additionally, the roles of IRF family members in different neurodegenerative diseases vary depending on disease-specific contexts. For example, IRF1 exacerbates inflammation in PD via VCAM-1 upregulation mediated by PINK1 mutants, whereas it aggravates demyelination injury in MS by regulating oligodendrocyte pyroptosis (caspase1-dependent) [73,86]. IRF3 promotes neuroinflammation in PD through the cGAS-STING pathway [76], yet it exhibits anti-inflammatory effects in AD and MS via the AKT/PI3K pathway [35,96] and suppresses inflammation in MS by modulating astrocyte miRNA [94]. Such functional contradictions underscore the need for precise and context-specific targeting strategies.

Currently, the understanding of the IRF family’s role in AD remains incomplete. Future research should focus on elucidating the precise mechanisms of IRF involvement in AD, such as by constructing conditional knockout/overexpression models for IRF members and integrating single-cell transcriptomics to dissect the spatiotemporal specificity of IRF signaling in neurons, microglia, and oligodendrocytes. Furthermore, an in-depth exploration of the interaction network among IRF family members could facilitate multi-pathway coordinated interventions. For instance, bifunctional molecules (e.g., curcumin derivatives combined with STING inhibitors) could be designed to simultaneously target the pro-inflammatory IRF3 pathway (via TLR4/STING) while enhancing its protective functions (via PI3K/Akt). Nanocarriers could enable lesion-specific delivery, thereby minimizing off-target effects. Developing selective IRF modulators would help overcome the therapeutic limitations imposed by functional redundancy and heterogeneity, driving the evolution of AD immunotherapy from “broad-spectrum anti-inflammation” toward “precise immune remodeling”. Preclinical and clinical evaluations of these strategies are essential to assess their efficacy. Targeting IRFs represents a promising strategy to address the complex interplay of inflammation and neurodegeneration in AD, potentially paving the way for more effective treatments.

## Figures and Tables

**Table 1:** Investigating the effect of IRF family in AD neuroinflammation.

IRFs	Phenotype	References
IRF1	IRF1 modulates pro-inflammatory and anti-inflammatory DAM.	[29]
	IRF1 transcriptionally regulates microglia together with LXRβ and CEBPα.	[29]
	IRF1 mediates inflammation-related processes of BIN1 and EZH2 inhibitor.	[30,31]
	Anti-inflammatory agents suppress the IRF1-related signaling pathway.	[32,33,34]
IRF3	IRF3 mRNA is highly elevated in AD brains.	[35]
	Bid, Tat, mtDNA positively and Scoparone negatively regulate TLR signaling to affect the pro-neuroinflammatory response of IRF3.	[36,37,38,39]
	cGAS-STING-IRF3 pathway in AD: cGAMP and IL-6 inhibit neuroinflammation, resveratrol protects cognition.	[40,41,42,43]
	Autophagy, pyroptosis, and IRF3 in AD: aggregation clearance, alleviation of inflammation and cognitive impairment by 3-MA and ZBP1 silencing.	[44,45]
IRF7	IRF7 mRNA is reduced in AD brains but upregulated in microglia during CNS injury.	[46,47,48]
	TREM2 R47H variant increases AD risk and neuroinflammation by enhancing IRF7 activation and type I interferon responses.	[49]
	IRF7 knockdown suppresses Aβ-induced IFN-α/β and p-STAT3 via TLR-Myd88 signaling, exacerbating AD pathogenesis.	[50]
	miR-146b deficiency increases IRF7 expression, which upregulates miR-146a, inhibits TLR4, and reduces NF-κB activation and neuroinflammation.	[51]
	TSG reduces IRF7 and neuroinflammation via cGAS-STING inhibition, with potential AD benefits.	[52]
	NIRL upregulates IRF7, promoting microglia phenotype shift, reducing brain damage and improving cognitive function.	[53]
IRF4 and IRF5	IRF5 promotes pro-inflammatory M1 microglial polarization and Aβ-driven neuroinflammation in AD, while IRF4 enhances anti-inflammatory M2 polarization and neuroprotection.	[54]
	Modulating the IRF5/4 ratio alleviates pathology via immune regulation and Aβ clearance, supported by microbiome-derived lipids and therapeutic interventions.	[48,55,56]
IRF8	IRF8 is upregulated in AD brains, associated with microglial activation markers, and is involved in TREM2-related AD pathogenesis.	[57]
	In AD transgenic Tg2576 mice, Aβ1-40 upregulates IRF8 via the JAK2/STAT1 pathway, driving microglial activation; silencing IRF8 reduces this effect.	[58]

## Data Availability

Not applicable.

## Abbreviations

AD: Alzheimer’s disease; IRFs: Interferon Regulatory Factors; DBD: DNA-binding domain; ISGs: IFN-stimulated genes; TREM2: triggering receptor expressed on myeloid cell 2; cGAS-STING: cyclic GMP-AMP synthase-stimulator of interferon genes; ER: endoplasmic reticulum; UPR: unfolded protein response; CNS: central nervous system; DAM: disease-associated microglia; BIN1: Bridging Integrator 1; LOAD: late-onset AD; EZH2: Enhancer of zeste homolog 2; PP: pheophytin α; CP: chlorophyll a; TLR: Toll-like receptor; Bid: BH3-interacting domain death agonist; Tat: trans-activator of transcription; TBK1: TANK-binding kinase 1; CXCR3: CXC chemokine receptor 3; TRIF: TIR-domain-containing adapter-inducing interferon-β; MyD88: myeloid differentiation primary response gene 88; CCH: chronic cerebral hypoperfusion; DHCR7: dehydrocholesterol-reductase-7; ZBP1: Z-DNA binding protein 1; PKR: protein kinase RNA-activated; TSG: Tetrahydroxy stilbene glucoside; PND: postoperative neurocognitive disorder; S/G: serine-glycine; PD: Parkinson’s disease; GCs: glucocorticoids; PINK1: PTEN-induced putative kinase 1; YAP1: Yes-associated protein 1; AIM2: absent in melanoma 2; MS: multiple sclerosis; EAE: experimental autoimmune encephalomyelitis; SNPs: single nucleotide polymorphisms; IDMF: Isosorbide di-(methyl fumarate); pDCs: plasmacytoid dendritic cells; ALS: amyotrophic lateral sclerosis; OPTN: optineurin; BMDM: bone marrow-derived macrophage; HD: Huntington’s disease; HTT: huntingtin; IKBKB: inhibitor of nuclear factor kappa B kinase subunit beta.
